# *mmi1* and *rep2* mRNAs are novel RNA targets of the Mei2 RNA-binding protein during early meiosis in *Schizosaccharomyces pombe*

**DOI:** 10.1098/rsob.180110

**Published:** 2018-09-26

**Authors:** Kaustav Mukherjee, Bruce Futcher, Janet Leatherwood

**Affiliations:** Department of Molecular Genetics and Microbiology, Stony Brook University, Stony Brook, NY 11790, USA

**Keywords:** Mei2, meiosis, pombe, lncRNA, Mmi1, Rep2

## Abstract

The RNA-binding protein Mei2 is crucial for meiosis in *Schizosaccharomyces pombe.* In *mei2* mutants, pre-meiotic S-phase is blocked, along with meiosis. Mei2 binds a long non-coding RNA (lncRNA) called meiRNA, which is a ‘sponge RNA’ for the meiotic inhibitor protein Mmi1. The interaction between Mei2, meiRNA and Mmi1 protein is essential for meiosis. But *mei2* mutants have stronger and different phenotypes than meiRNA mutants, since *mei2Δ* arrests before pre-meiotic S, while the meiRNA mutant arrests after pre-meiotic S but before meiosis. This suggests Mei2 may bind additional RNAs. To identify novel RNA targets of Mei2, which might explain how Mei2 regulates pre-meiotic S, we used RNA immunoprecipitation and cross-linking immunoprecipitation. In addition to meiRNA, we found the mRNAs for *mmi1* (which encodes Mmi1) and for the S-phase transcription factor *rep2*. There were also three other RNAs of uncertain relevance. We suggest that at meiotic initiation, Mei2 may sequester *rep2* mRNA to help allow pre-meiotic S, and then may bind both meiRNA and *mmi1* mRNA to inactivate Mmi1 at two levels, the protein level (as previously known), and also the mRNA level, allowing meiosis. We call Mei2–meiRNA a ‘double sponge’ (i.e. binding both an mRNA and its encoded protein).

## 1. Introduction

Mei2 is an RNA-binding protein required for initiating pre-meiotic DNA synthesis and meiosis in *Schizosaccharomyces pombe*. Its only known target is meiRNA, a long non-coding RNA encoded by the *sme2* gene [1]. Mei2 has two RNA recognition motifs (RRMs), and the C-terminal RRM is required for meiosis initiation [2]. During vegetative growth, Mei2 is inactivated and the meiotic program kept off because the Pat1 kinase phosphorylates Mei2 [3], inducing its degradation by the proteasome [4]. Signals that induce meiosis inhibit Pat1 kinase activity, allowing stabilization and accumulation of Mei2. Mutants of Mei2 that cannot be phosphorylated and degraded initiate meiosis without nitrogen starvation [3].

meiRNA is an example of a long non-coding RNA (lncRNA), which have diverse functions in eukaryotes. They range from transcriptional regulation in the nucleus, and regulation of chromatin structure [5], to posttranscriptional gene regulation [6]. lncRNAs have also been implicated in human diseases and cancer [7,8]. In fission yeast, lncRNAs play important regulatory roles in cell differentiation [9], drug tolerance [10] and phosphate metabolism [11–13].

In part—but only in part—Mei2 allows meiosis because it binds meiRNA [1,2]. The Mei2 protein localizes at a specific locus in the nucleus during meiosis, forming a ‘dot’ [1] even before the karyogamy stage of meiosis [2]. The meiRNA binds to the Mei2 protein, and is required to form the Mei2 dot. The Mei2 protein–meiRNA complex is formed at the locus of the *sme2* gene, which encodes the meiRNA [14], but almost certainly not any protein [1]. Mei2 protein alone can apparently shuttle between the cytoplasm and nucleus, but binding of meiRNA traps Mei2 protein in the nucleus during meiosis [15,16]. However, adding an NLS to Mei2 protein allows it to localize to the nucleus and form a dot and promote meiosis even in the absence of meiRNA [2]. Importantly, the C-terminal RRM of Mei2 is critical for function even when the meiRNA is absent: addition of an NLS to a Mei2 mutant protein lacking this C-terminal RRM does not enable it to suppress the loss of meiRNA [2]. Thus, Mei2 protein is required in the nucleus to initiate meiosis, and this function requires its RNA-binding ability, not just nuclear localization.

The significance of the Mei2 dot was revealed when the Mei2–meiRNA complex was found to colocalize with another RNA-binding protein, Mmi1, in the nucleus during meiosis. Mmi1 is a crucial inhibitor of meiosis. During vegetative growth, Mmi1 binds to transcripts required for meiotic entry and for early meiosis, and targets them for degradation via the exosome, keeping meiosis off [17–20]. Mmi1 also recruits the Ccr4–Not complex leading to Mei2 ubiquitination by Mot2, and turnover of Mei2 protein [21]. Upon nitrogen starvation and meiotic initiation, the Mei2–meiRNA complex sequesters Mmi1 protein in the nucleus, thus allowing the mRNAs targeted by Mmi1 to escape degradation and be translated in the cytoplasm, thus initiating meiosis [17]. Because Mei2 inhibits Mmi1 protein (by sequestering it in a dot in the nucleus), while Mmi1 inhibits Mei2 (by inducing turnover), these two proteins form a double negative feedback loop, which can act as a meiotic ‘switch’.

Mmi1 binds to the hexanucleotide motif T(T/C/G)AAAC on its target RNAs [19,22]. Strikingly, the long, meiotic form of meiRNA has 23 of these Mmi1 motifs [23]. These motifs bind and titrate Mmi1 to keep it localized to the *sme2* locus in the nucleus during meiosis [19,23]. Thus, meiRNA is a decoy RNA, or sponge RNA, which titrates out and effectively inactivates Mmi1 protein [23]. During vegetative growth, a shorter form of meiRNA lacking most of these Mmi1 motifs is made, and this form of meiRNA is degraded by the exosome [23]. This partly explains why meiRNA is unable to sequester Mmi1 during vegetative growth. Mei2 binds to the 5′ end of meiRNA [23].

Although the results cited above show that the Mei2–meiRNA complex promotes meiosis at least partly by titrating out Mmi1 protein, this is not the whole story. Several workers have shown that the *mei2* mutant has a stronger and qualitatively different meiotic phenotype than the *sme2* (meiRNA) mutant. For instance, upon nitrogen starvation, the *mei2Δ* mutant is defective in pre-meiotic S-phase (and, of course, in meiosis). However, the *sme2Δ* mutant performs pre-meiotic DNA synthesis, but cannot undertake meiosis [1]. The ts allele *mei2-33*, an allele where binding of the meiRNA is defective at restrictive temperature, mimics *sme2Δ*, and performs pre-meiotic DNA synthesis, but not meiosis [1]. Finally, the *mei2-644A* mutant, bearing a mutation in the C-terminal RRM, is phenotypically similar to the *mei2Δ* allele, in that it is defective in pre-meiotic DNA synthesis (and meiosis) [1]. This led Watanabe & Yamamoto [1] to suggest ‘Mei2 functions at least twice, prior to pre-meiotic DNA synthesis and prior to meiosis I, by coupling with different RNA partners’. That is, they suggest Mei2 must have two targets, one responsible for allowing pre-meiotic S and a second (presumably meiRNA) responsible for meiosis. Subsequent work showed that if the Mei2 protein is provided with a nuclear localization signal, it can promote meiosis even in the absence of meiRNA (i.e. it suppresses a *sme2/sme2* null mutant) [2]. However, Mei2-NLS can only suppress *sme2/sme2* if the C-terminal RRM of Mei2 is intact, suggesting there may be one or more additional RNA targets for promoting meiosis, and leading Yamashita *et al*. [2] to suggest that ‘Mei2p may have to interact with one or more RNA species in the nucleus in order to promote meiosis I.’ Shichino *et al*. [23] concluded that over-expression of meiRNA does not fully inactivate Mmi1, likewise suggesting Mei2 may have an additional, meiRNA-independent mechanism for inactivation of Mmi1. These various observations that the phenotype of *mei2* is stronger and different from the phenotype of *sme2Δ* (meiRNA) all point to the possibility that Mei2 might bind additional RNA targets. Potentially there is a novel RNA target specifically involved in pre-meiotic S-phase, and possibly an additional novel target helping to inhibit Mmi1 activity, or in otherwise promoting meiosis. This idea motivated us to look for novel RNA-binding partners of Mei2, using techniques such as RIP-CHIP [24] and CLIP-Seq [25] to isolate Mei2, and identify any novel associated RNAs. Strikingly, in addition to meiRNA, we found the *mmi1* mRNA as a target of Mei2, suggesting that Mei2 can inhibit Mmi1 activity independently at two levels, the mRNA level and the protein level. In addition, we found the mRNA for the S-phase transcription factor *rep2*, a factor important for transcription of vegetative S-phase transcripts rather than meiotic transcripts. The binding of *rep2* mRNA could potentially explain why *mei2Δ*, but not *sme2Δ*, fails at pre-meiotic DNA synthesis.

## 2. Material and methods

### 2.1. Yeast strains, media and growth conditions

*Schizosaccharomyces pombe* culture methods were as described in [26]. When required, cells were grown in Edinburgh Minimal Media (EMM) with supplements. For transformation, cells were grown in liquid YES at 32°C overnight and then diluted in equal volume of fresh YES media. Cells were grown another 3 h before harvesting. For nitrogen starvation and sporulation, sporulation agar (SPA) and malt extract (ME) media were used [26]. For immunoprecipitation experiments using h^90^ strains (JLP14, JLP1760 and JLP1772), cells were grown at 32°C on YES up to OD_600_ = 0.6–0.8. Then they were washed and inoculated into ME media for 4–5 h at 23°C. All strains used in this study are listed in electronic supplementary material, table S4.

### 2.2. Construction of TAP-tagged Mei2 and Msa1 strains

The TAP tag cassette was amplified from plasmid pFA-6a-TAP-Kan with 300 nucleotides 5′ and 3′ sequences complementary to either Mei2 C-terminus or Msa1 C-terminus. This was transformed using the protocol by [27]. Kanamycin-resistant transformants were selected on YES-G418 plates, and TAP tag integration was confirmed by colony PCR and sequencing. Primers used are listed in electronic supplementary material, table S4.

### 2.3. Western blot

Cells were lysed according to the procedure mentioned in [26] and equal amounts of protein from each sample were resolved by 10% SDS-PAGE. Mei2-TAP and Msa1-TAP was detected by chemiluminescence using a 1 : 5000 dilution of peroxidase anti-peroxidase (PAP) antibody.

### 2.4. Native RNA immunoprecipitation

Cells were collected in ice, washed with cold lysis buffer, flash-frozen in liquid nitrogen and stored at −80°C. RNA immunoprecipitation (RIP) was done according to the protocol in [28] with the following modifications. All steps were performed in a cold room on ice. Protease inhibitors leupeptin, pepstatin, bestatin and aprotinin were added to a final concentration of 2 µg ml^−1^ of lysis buffer. PMSF was added to a final concentration of 1 mM and 100 U ml^−1^ of SuperaseIn (Ambion) was also added to lysis buffer. Cells were lysed by bead beating using 0.7 mm Zirconia beads in a Fast-Prep at maximum speed for 2 pulses of 20 s and incubation for 5 min on ice in between pulses. 20 µl of lysate was frozen in liquid nitrogen for western, and 50 µl was stored in liquid nitrogen as input. IgG-Agarose beads (Sigma) were used for immunoprecipitation at 4°C for 2 h. Beads were washed for a total of nine washes, three times in lysis buffer, and six times in wash buffer containing NP-40, glycerol, heparin, RNase and protease inhibitors. RNA was eluted from the beads using 1% SDS in 1X TE with SuperaseIn. RNA was purified by phenol:chloroform extraction and sodium acetate and ethanol precipitation.

### 2.5. Microarray

Microarray labelling and hybridization was performed using a two-colour Quick Amp Labeling Kit (Agilent). Labelled cDNAs were hybridized onto custom-made Agilent 2 × 105 K comparative genome hybridization (CGH) arrays for *S. pombe* (Agilent). Immunoprecipitated RNAs were labelled with Cy3. The CGH array had 50 nucleotide probes with 50 nucleotide gaps on each strand. Therefore, the genome coverage is roughly 50%. Normalized Cy5 intensities were visualized on Integrated Genome Browser (IGB) [29]. The RIP-CHIP data are available in electronic supplementary material as processed files in ‘.egr’ format for viewing in IGB. The raw data including array design are available in GEO (GSE114299).

### 2.6. UV crosslinking and immunoprecipitation

Cross-linking immunoprecipitation of Mei2-TAP and Msa1-TAP were done according to [30] with some modifications. Cells were UV cross-linked using Stratalinker with power 100 and default time settings. Cells were lysed exactly as in RIP (above). Lysates were sonicated using a Misonix sonicator at 80 amplitude for 30 s. Immunoprecipitation, washes and elution were done as in RIP, but with different buffers as in [30]. Following elution, 1 µl of 20 mg ml^−1^ proteinase K was added to the eluates and incubated for 1 h at 42°C. RNA was extracted by phenol:chloroform and precipitated using sodium acetate and ethanol.

### 2.7. RNA sequencing of immunoprecipitated RNA and data analysis

Immunoprecipitated RNA was DNase treated, and libraries for Illumina sequencing were made using a Nugen Ovation Universal RNA-Seq system. cDNA was made using a random-primed dUTP method to preserve strand-specificity. cDNA was sonicated using a Misonix 3000 sonicator at 60 amplitude for 10 min on ice. Libraries were PCR-amplified for 12 cycles. The quality of libraries was checked using Bioanalyzer and Qubit, and sequenced on Illumina MiSeq by paired-end sequencing. Reads were mapped to *S. pombe* reference transcriptome version ASM294 v. 2.26 using STAR [31]. Discordant mapping was not allowed and only annotated splice junctions were mapped. Alignments in bam format were sorted and indexed using Samtools and visualized on Integrative Genomics Viewer [32]. Deeptools ‘bamCoverage’ [33] was used to convert bam files to strand-specific bigwig files to visualize pileups at specific loci on IGV.

Peak-calling, enrichment analysis and peak annotation was done using Homer [34]. ‘Histone-mode’ was enabled in Homer to allow for wide peaks. Mei2-specific RNA targets were determined by differential peak calling against CLIP-Seq tags from untagged, and Msa1-TAP separately (electronic supplementary material, table S1). Highly significant Mei2 targets were determined by selecting the top 20 significant peaks (by *p*-value) from Mei2 versus untagged and Mei2 versus Msa1, and taking the intersection of the two sets. This provided us with 10 highly significant CLIP-Seq targets of Mei2 (electronic supplementary material, table S2). Msa1-specific targets were determined by differential peak calling against untagged CLIP-Seq tags (electronic supplementary material, table S3). Our CLIP-Seq data including the bigwig and peak files are available in GEO, accession code: GSE112117.

For enrichment analysis in figure 3, Homer was used to calculate the tag counts for 5 nucleotide bins over the entire length of the target RNA. These were then normalized to the percentage length of each RNA. Subsequently these were hierarchically clustered with Euclidian distance as the similarity metric and simple linkage using Cluster v. 3.0 [35]. The clusters were visualized by Java Treeview [36].

### 2.8. Quantitative RT-PCR

Input and co-immunoprecipitated RNAs were used to make first strand cDNA using Superscript RT III (Invitrogen Life Technologies) according to manufacturer's instructions. cDNA was used for quantitative PCR using Roche LightCycler 480 SYBR Green Master kit with gene specific primers (electronic supplementary material, table S4).

## 3. Results and discussion

### 3.1. Expression of Mei2 after nitrogen starvation and in early meiosis

We wished to identify novel RNA targets of Mei2 in meiosis, and for that purpose it was important to know at what times in meiotic time-courses Mei2 was being highly expressed. We used western blotting to assay Mei2 protein levels at various times after nitrogen starvation (which was used to initiate meiosis). We added a TAP-tag to the C-terminus of Mei2 in an h^90^ strain and allowed it to sporulate in nitrogen-deficient ME medium. We found that the Mei2-TAP strain sporulated as efficiently as wild-type (data not shown), indicating that addition of a TAP tag to Mei2 does not affect sporulation. Next, we did a time course and looked for Mei2 expression at different times after nitrogen starvation. We found two bands roughly corresponding to the size of full-length Mei2-TAP from 3 h to 6 h after nitrogen starvation (electronic supplementary material, figure S1A). We found by microscopy that, during these times, most cells had conjugated (data not shown). Mei2-TAP levels were higher at the 3 h and 6 h data points than at other times; this could indicate regulation of Mei2 abundance, but could also reflect asynchronous meiosis. We noticed multiple bands shorter than full-length Mei2-TAP (electronic supplementary material, figure S1A). These were probably degradation products of Mei2 protein.

### 3.2. Native RNA immunoprecipitation and UV cross-linking and immunoprecipitation of Mei2-TAP

To identify RNAs associated with the Mei2 RNA-binding protein during early meiosis, we used pooled samples with high Mei2 expression (4–6 h after nitrogen starvation) to immunoprecipitate Mei2-TAP with agarose-coupled IgG beads. We tested Mei2-TAP enrichment after immunoprecipitation using western blotting with a PAP antibody and found that a significant proportion of cellular Mei2-TAP was associated with the IgG beads (electronic supplementary material, figure S2A). To stabilize Mei2–RNA interactions, we used UV cross-linking and immunoprecipitation (CLIP) of Mei2-TAP according to the protocol from [37]. Many groups that study RNA–protein interactions have observed that some RNAs co-immunoprecipitate promiscuously with most RNA-binding proteins (Juan Mata 2011, personal communication). To control for such noise, we added a TAP tag to the C-terminus of another RNA-binding protein, Msa1, and found that Msa1-TAP was expressed throughout meiosis (electronic supplementary material, figure S1B). Both Mei2 and Msa1 had some level of protein degradation in meiosis, despite the addition of protease inhibitors (electronic supplementary material, figure S1C).

We performed CLIP on nitrogen starved h^90^ cells which were Mei2-TAP (experiment), or Msa1-TAP (control), or untagged (control). The lysates were lightly sonicated after cross-linking to break up complexes. Analysis by western blotting showed strong association of Mei2-TAP and Msa1-TAP with the IgG beads after CLIP (electronic supplementary material, figure S2B).

**Figure 1.**
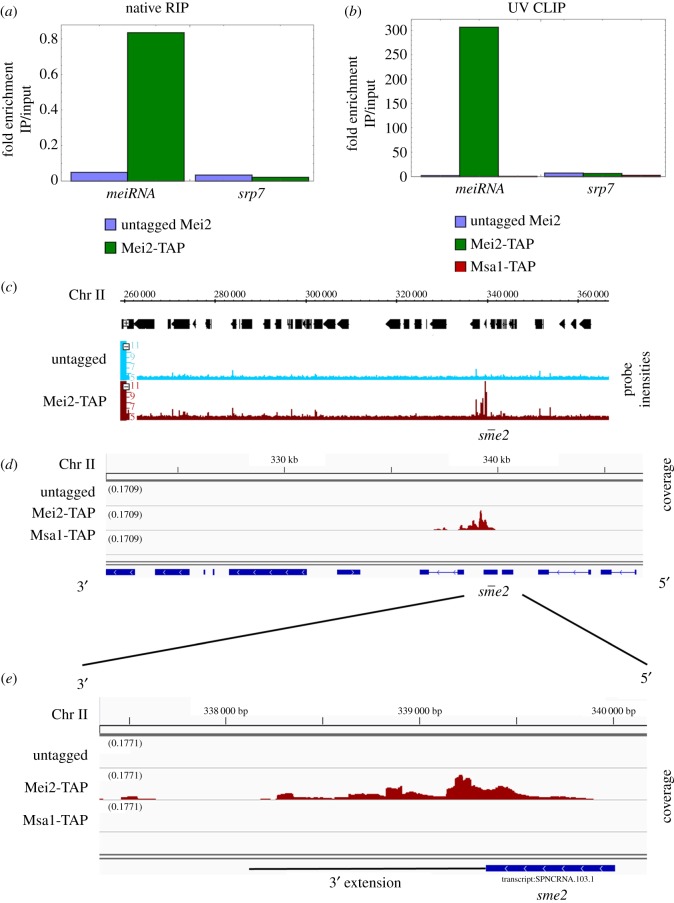
*meiRNA* enrichment after Mei2-TAP immunoprecipitation. (*a*) *meiRNA* enrichment after native RNA immunoprecipitation (RIP) of Mei2 shown by qRT-PCR. The *y*-axis shows log2 fold enrichment. *srp7* is shown as a specificity control. (*b*) *meiRNA* enrichment after UV cross-linking immunoprecipitation (CLIP) by qRT-PCR is shown. *srp7* is shown as a specificity control. (*c*) Integrated Genome Browser view of the *sme2* locus after RIP-CHIP. (*d*) Integrative Genomics Viewer snapshot of the *sme2* locus after CLIP-Seq. (*e*) CLIP-Seq coverage reads at the *sme2* locus. The *y*-axis scale is normalized for all samples in (*c–e*).

We purified RNAs that co-immunoprecipitated with Mei2-TAP after RIP, and Mei2-TAP and Msa1-TAP after CLIP. Since meiRNA is a known target of Mei2, we used qRT-PCR to test for meiRNA enrichment and found that it was highly enriched after both RIP and CLIP experiments (figure 1*a*,*b*). The fold enrichment was significantly higher after CLIP (figure 1*b*). To ensure specificity, we tested for enrichment of the control mRNA *srp7*, which should not bind Mei2, and found that it was not enriched (figure 1*a*,*b*). meiRNA was not enriched in the control CLIP experiments with Msa1-TAP, indicating that meiRNA binds specifically to Mei2 in these experiments (figure 1*b*).

### 3.3. Novel RNA-binding partners of Mei2 discovered by RIP-CHIP and CLIP-Seq

Since the specific enrichment of meiRNA suggested that the RIP experiment was successful, we looked for novel targets of Mei2 by hybridizing the extracted RNAs from RIP to a microarray (see methods). We used a tiling array chip with 50 nucleotide strand-specific probes (see Methods). We searched the genome for enriched regions using the Integrated Genome Browser and found 13 RNAs (including meiRNA) specifically enriched in the Mei2-TAP experiment versus untagged control (table 1 and figure 1*c*).

**Table 1.** RNA targets of Mei2.

gene	function
*Mei2 targets*	
*sme2*	ncRNA acts as Mmi1 sponge
*mmi1*	meiotic RNA turnover
*rep2*	MBF Transcription Factor
*mug110*	unknown
*SPACUNK4.17*	NAD binding dehydrogenase family protein
*SPBP23A10.11c*	cell wall biosynthesis
*RIP-CHIP only*	
*SPBC32F12.07c*	ubiquitin E3 ligase
*yox1*	MBF regulatory subunit
*efc25*	Ras1 guanyl-nucleotide exchange factor
*SPCC1223.14*	chorismate synthesis
*SPCC757.12*	alpha amylase homologue
*snoU14*	unknown
*but2*	protein neddylation with Uba3
*CLIP-Seq only*	
*SPAC11D3.01c*	unknown
*rbd4*	rhomboid family protease
*tif51*	translation elongation and termination factor eIF5A (predicted)
*ubc7*	Hrd1 ubiquitin E2 ligase

As an independent assay for novel RNAs, we also identified Mei2 and Msa1 targets from CLIP. For this, RNAs were assayed by reverse transcription and cDNA sequencing. We mapped the sequenced reads to the *S. pombe* transcriptome, visualized the alignments using IGV, and computed pileups at target loci. Exactly as in the RIP experiments (above) we found that meiRNA was highly enriched in the Mei2-TAP CLIP-Seq but not in the control Msa1-TAP CLIP-Seq (figure 1*d*). We used the peak-calling and annotation program Homer to analyse the genome for additional Mei2 targets (electronic supplementary material, table S1). We classified RNAs as Mei2 CLIP-Seq targets only if those RNAs immunoprecipitated in the Mei2-TAP strain but not in the control untagged strain or the control Msa1-TAP strain (electronic supplementary material, table S2, see methods).

Furthermore, to guard against the possibility that Mei2 might bind non-specifically to highly expressed meiotic RNAs, we looked at the mRNAs for many highly expressed, early meiotic genes, but none of these mRNAs was enriched in either experiment. Two examples, *rec8* and *mei4,* are shown in electronic supplementary material, figure S3A,B.

**Figure 2.**
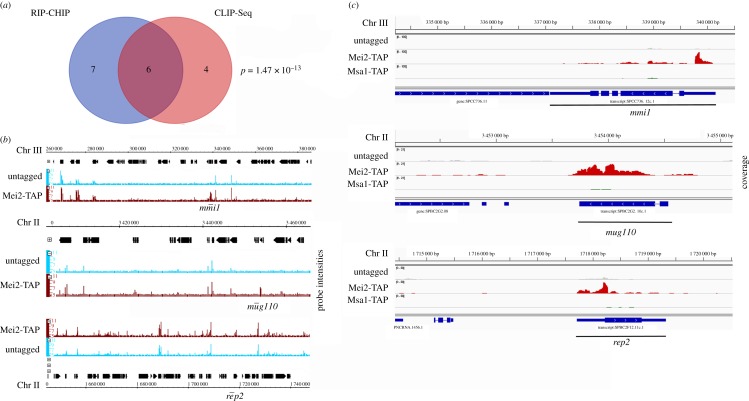
RNA partners of Mei2 in early meiosis with known roles in meiosis. (*a*) Venn diagram showing number of significant targets obtained by Mei2-TAP RIP-CHIP and CLIP-Seq. 6 RNA targets of Mei2 were enriched in both experiments. (*b*) Probe intensities from RIP-CHIP for important Mei2 targets *mmi1, rep2* and *mug110*. (*c*) CLIP-Seq coverage from Mei2-TAP and Msa1 at the *mmi1, rep2*, and *mug110* loci. The *y*-axis scale is normalized for (*b*) and (*c*).

There were 13 targets of Mei2 as determined by RIP-CHIP (table 1, figures 1*c* and 2*b*; electronic supplementary material, figures S4 and S5), and 10 targets as determined by the independent CLIP-SEQ experiment (electronic supplementary material, figures S6 and S7). Strikingly, six of these targets (including *meiRNA*) overlapped between the two independent experimental approaches (*p*-value ∼ 10^−13^) (figure 2*a*). We considered these as high-confidence Mei2 targets (figure 2*b*,*c*; electronic supplementary material, figures S4 and S6). The functions of these targets are summarized in table 1. Two of the five novel targets, *mmi1* and *rep2* (figure 2*b*,*c*), have well characterized biological roles in controlling gene expression and meiosis.

The *mmi1* mRNA has obvious relevance, since the Mmi1 protein inhibits meiotic entry, and since the Mmi1 protein is already known to be a target of the Mei2–meiRNA complex. Our finding here suggests that Mei2 is inhibiting Mmi1 function at two levels, the protein level (as previously shown) and also, separately and independently, the mRNA level. This could lead to a much more profound inhibition of total Mmi1 activity than if the Mei2–meiRNA complex were targeting Mmi1 protein only. Interestingly, we observe that the *mmi1* primary transcript has nine consensus sequence binding sites for Mmi1 protein (T(TCG)AAAC), and the mature mRNA has seven. This suggests that there could be negative feedback of Mmi1 protein on *mmi1* mRNA, or, perhaps more speculatively, that *mmi1* mRNA itself is a sponge RNA for its own protein. Interestingly, CRAC analysis suggests that Mmi1 protein may bind to its own transcript during vegetative growth [38], but there is no evidence that Mmi1 targets its own transcript for destruction, somewhat supporting the view that *mmi1* RNA could be a sponge for its own protein.

Rep2 is a component of the MBF transcription factor complex [39]. The MBF complex comprises Res1, Res2, Cdc10 and Rep2 in vegetative cells, and is responsible for transcription of S-phase genes during vegetative growth, including many genes needed for DNA replication [39–43]. However cellular requirements during vegetative S-phase, and during pre-meiotic S-phase, are not precisely the same, and *rep2* RNA expression is reduced during nitrogen starvation [39]. Furthermore, overexpression of Rep2 blocks G1 arrest following nitrogen starvation, whereas depletion of Rep2 facilitates G1 arrest after nitrogen starvation [39]. This indicates that Rep2 promotes vegetative S-phase, and in that sense inhibits meiotic entry. During meiosis, the meiotic MBF transcription factor Rep1 probably replaces Rep2 in the MBF complex, since Rep1 is critical for transcribing a subset of pre-meiotic S-phase genes [43–46]. Sequestration of *rep2* mRNA in the nucleus by the Mei2–*meiRNA* complex could aid in replacement of Rep2 by Rep1 in the MBF complex, thus promoting pre-meiotic S.

*mug110* is a *m*eiosis *u*pregulated *g*ene of unknown function. Previously *mug110* mRNA was shown to bind another RNA-binding protein, Crp79 [47]. *mug110* was not enriched in Msa1-TAP CLIP-Seq (figure 2*c*; electronic supplementary material, figure S6) suggesting that it binds specifically to Mei2 in early meiosis. *mug110* is a meiosis upregulated gene, but the *mug110Δ* strain has no obvious meiosis or sporulation defects [48]. We made a *mug110Δ mei2Δ* double mutant and found that the phenotype of *mug110Δ mei2Δ* was indistinguishable from *mei2Δ*; the double mutant did not initiate meiosis (data not shown).

*SPACUNK4.17* (electronic supplementary material, figure S4) is a NAD dehydrogenase family protein with an unknown function in *S. pombe.* In a recent screen for mutants that affect sexual reproduction, *SPACUNK4.17Δ* was shown to have cell-fusion defects, low mating efficiency and multi-septate cells [49]. We have noticed cell fusion defects in *mei2Δ* cells. The phenotypes of *SPACUNK4.17Δ* are either mild to moderate, but this gene could play a yet undiscovered role in early meiosis.

Finally, *SPBP23A10.11c* (electronic supplementary material, figure S4) is poorly characterized. It is an orthologue of *S. cerevisiae TOS1*, which encodes a cell wall protein under the control of the SBF/MBF transcription factor. Its RNA level goes down in response to nitrogen starvation and meiosis [50].

Some RNAs co-immunoprecipitated with Mei2 in only RIP-CHIP but not CLIP-Seq or vice versa (table 1). We did not classify these two sets of RNAs as high-confidence Mei2 targets (electronic supplementary material, figures S5 and S7). A few RNAs were enriched in both Mei2-TAP and Msa1-TAP CLIP-Seq but not in untagged controls (not shown). Both Mei2 and Msa1 are meiotic RNA-binding proteins so they may have common targets. However, some RNAs co-precipitate promiscuously in RNA IP experiments and we considered this to be because of non-specific interactions between RNAs and RNA-binding proteins.

### 3.4. Do the novel targets explain why mei2 mutants have stronger phenotypes than meiRNA?

Our investigation began with the observation that a *mei2* mutant has a stronger and different meiotic phenotype than a meiRNA mutant. This suggested that the Mei2 protein might target additional RNAs. The five novel targets here, and in particular *mmi1* mRNA and *rep2* mRNA, are good candidates for such additional targets. However, the experiments required to prove this point are somewhat subtle: it is not sufficient to simply delete a candidate such as *mmi1*, as that removes both the mRNA and the protein, and it is already clear that an *mmi1* null mutant has an extreme phenotype. Ideally, one would identify the residues in each mRNA allowing that mRNA to bind to Mei2; then mutate those residues, creating a mutant mRNA that fails to bind Mei2, and ask whether this (in combination with a *sme2* deletion) phenocopies a *mei2* deletion. To this end, we have searched for sequence commonalities between the RNAs that bind Mei2 (e.g. using MEME), but so far without success. At present, the sequence determinants allowing binding to Mei2 remain unknown.

In wild-type meiotic cells, Mei2 forms a dot at the *sme2* locus. In a *sme2Δ* mutant, there is no dot. However, when an NLS is added to Mei2, then a dot is formed even in a *sme2Δ* mutant [2]. It will be interesting to see if this dot has any specific localization, and in particular whether it might be at any of the loci we have identified as expressing additional targets of Mei2 (*mmi1*, *rep2*, *mug110*, etc.)

### 3.5. Mei2 may have a preference for binding 5′ regions of target RNAs

meiRNA is a long non-coding RNA that is critical for meiosis. During meiosis, meiRNA has a 3′ extended form, and Mei2 binds to the 5′ end [23], while Mmi1 binds to the 3′ region. We found that the 3′ extended meiotic form of meiRNA co-immunoprecipitated with Mei2-TAP (figure 1*e*)*.* However, more reads aligned to the 5′ end of meiRNA (figure 1*e*), so our results are consistent with a previous study [23] showing that Mei2 binds to the 5′ end of meiRNA.

Strikingly, the 5′ UTR regions of *mmi1* and *rep2* mRNAs were enriched in Mei2-TAP CLIP-Seq (figure 2*c*). This could indicate that Mei2 binds specifically to the 5′ UTR of *mmi1* and *rep2.* It is also possible that the sequencing reads are restricted to the 5′ UTR due to an artefact of library preparation using random hexamers. However, we did not find a 5′ enrichment of other Mei2 target RNAs, e.g. *mug110 and SPACUNK4.17* (figure 2*c*; electronic supplementary material, figure S6), suggesting that the observed 5′ enrichment is not coincidental.

**Figure 3.**
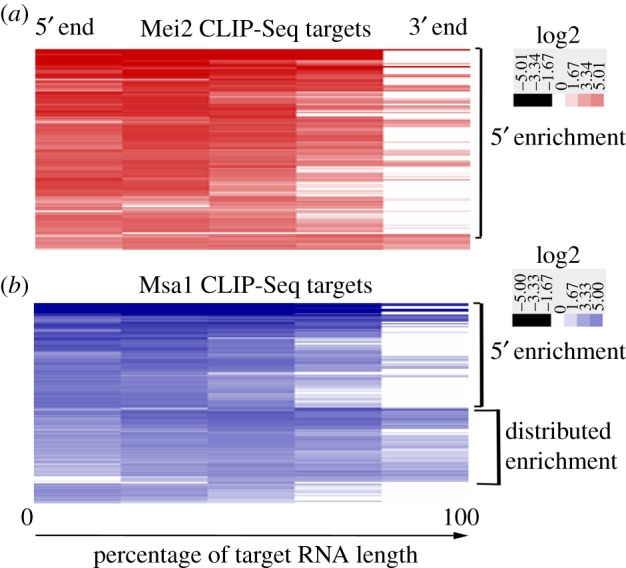
Enrichment profile along RNA length of Mei2 and Msa1 targets. (*a*) Enrichment profile of significant targets of Mei2 from CLIP-Seq determined by Homer. Each column in the heatmap shows enrichment that was calculated for a 5-nucleotide bin. Each row of the heatmap shows enrichment from 5′ to 3′ along the length of one particular Mei2 RNA target, normalized by length. (*b*) Enrichment profiles along length of Msa1 CLIP-Seq targets.

To test if Mei2 has a binding preference on its RNA targets, we used Homer to calculate the enrichment of all Mei2 CLIP-Seq targets genome-wide, over the entire length of each RNA. We then clustered the global enrichment profile of Mei2 targets (figure 3*a*). We found that predominantly (about 60%) 5′ regions of Mei2 RNA targets were enriched, including some that had high enrichment of extreme 5′ ends (figure 3*a*). This strongly suggests that Mei2 has a preference for binding 5′ ends of its target RNAs, and in some cases the 5′ UTR. We also made a similar enrichment profile for Msa1 CLIP-Seq targets (electronic supplementary material, table S3), and found that about half of them had 5′ region enrichment, and the other half had enrichment distributed across the RNA (figure 3*b*). This confirmed that not all RNAs we recovered from CLIP-Seq had 5′ enrichment, so this enrichment was not an artefact of sequencing library preparation.

## 4. Conclusion

**Figure 4.**
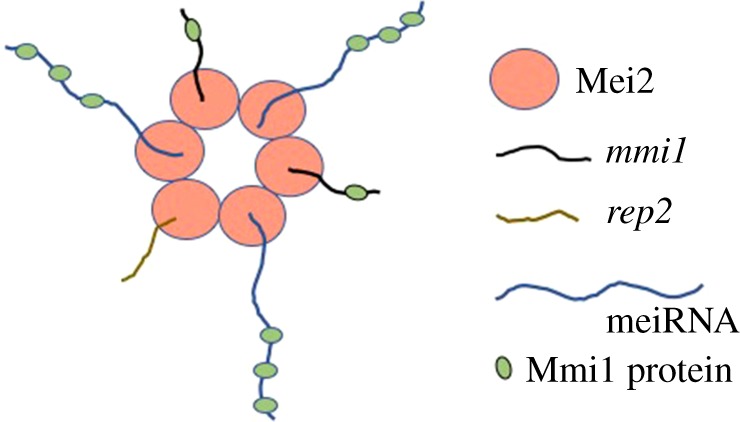
Mei2 titrates out inhibitors of meiosis. During early meiosis Mei2 binds to meiRNA, which binds Mmi1 protein, thus sequestering Mmi1. Mei2 also binds *mmi1* mRNA, and probably prevents synthesis of new Mmi1 protein. Mei2 also binds *rep2* mRNA, and probably prevents synthesis of new Rep2 protein. Titration of these three meiotic inhibitors (Mmi1, *mmi1* mRNA, and *rep2* mRNA) facilitate initiation of meiosis. The mechanism by which Mei2 forms a dot (here shown as a speculative Mei2–Mei2 protein–protein interaction) is unknown. Also unknown is whether the Mmi1 binding sites on the *mmi1* mRNA participate in the titration of Mmi1 protein.

In summary, Mei2 protein binds to the meiRNA*,* which acts as a sponge for Mmi1 protein in the nucleus during early meiosis [23]. Mmi1 is inhibitory towards meiosis, and *mmi1* mutants attempt meiosis even without nitrogen starvation [17]. We show here that in early meiosis, Mei2 not only binds to the Mmi1 protein, but also to the *mmi1* mRNA. We suggest this interaction prevents *mmi1* mRNA export to the cytoplasm for translation and inhibits synthesis of new Mmi1 protein. Thus, we propose a model where Mei2 simultaneously inhibits the action of *mmi1* at two levels (the protein level and the transcript level), resulting in a deeper inhibition of Mmi1 activity than could be achieved by inhibition at one level alone (figure 4). Interestingly, we have also observed that the *mmi1* mRNA contains multiple (7 to 9) binding sites for Mmi1, so *mmi1* mRNA could act as a sponge RNA for its own protein. While Mei2 inhibits Mmi1 function at two levels, Mmi1 also inhibits Mei2 via proteolysis [21], creating a double-negative feedback loop that could act as a meiotic switch.

Mei2 also binds to *rep2* mRNA, possibly preventing the synthesis of new Rep2 protein (figure 4). In vegetative cells, Rep2 is a component of the MBF transcription factor, but in response to nitrogen starvation, expression of Rep1 increases, and it replaces Rep2 in the pre-meiotic MBF complex. We suggest this replacement is enhanced by the inhibition of Rep2 synthesis caused by the sequestration of *rep2* mRNA by Mei2. Thus, in a *mei2* null mutant, at least some MBF complexes would continue to contain Rep2, and this could help explain why *mei2* mutants fail to accomplish pre-meiotic DNA synthesis. Of course, the profound inhibition of Mmi1 function by Mei2 acting at two levels could also contribute to pre-meiotic S.

## Supplementary Material

Supplementary Figures

## Supplementary Material

Supplementary Legends

## Supplementary Material

RIP-CHIP_egrfiles

## Supplementary Material

Supplementary Table 1 - Mei2 peaks

## Supplementary Material

Supplementary Table 2 - Mei2 CLIP targets

## Supplementary Material

Supplementary Table 3 - Msa1 peaks

## Supplementary Material

Supplementary Table 4 - Strains and primers

## Data accessibility

CLIP-Seq data are available in GEO, accession code: GSE112117 (reviewer token: ‘wbyrcucolvonbsh’). RIP-CHIP data are available as raw and processed text files in GEO, accession code: GSE114299 (reviewer token: ‘mdgvaqkorfatjsz’).
