# Supplementary material for: mmi1 and rep2 mRNAs are novel RNA targets of the Mei2 RNA-binding protein during early meiosis in Schizosaccharomyces pombe
Source: Open Biol. 2018 Sep 26;8(9):180110. doi: 10.1098/rsob.180110 (PMC6170507; doi:10.1098/rsob.180110)

Supplementary Figure S1

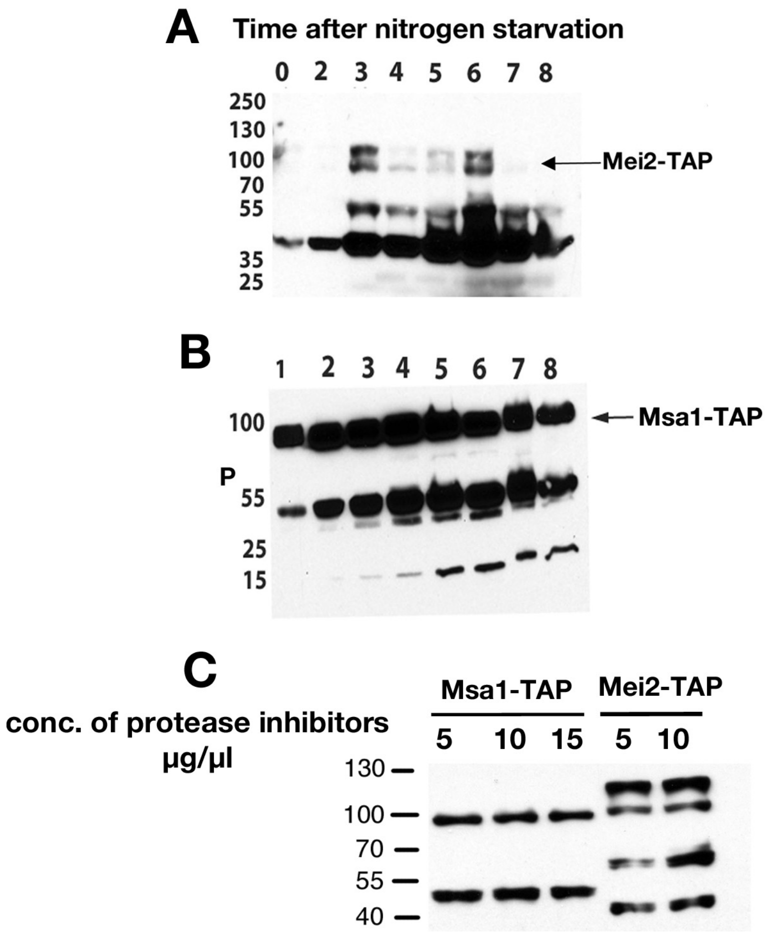

Supplementary Figure S2

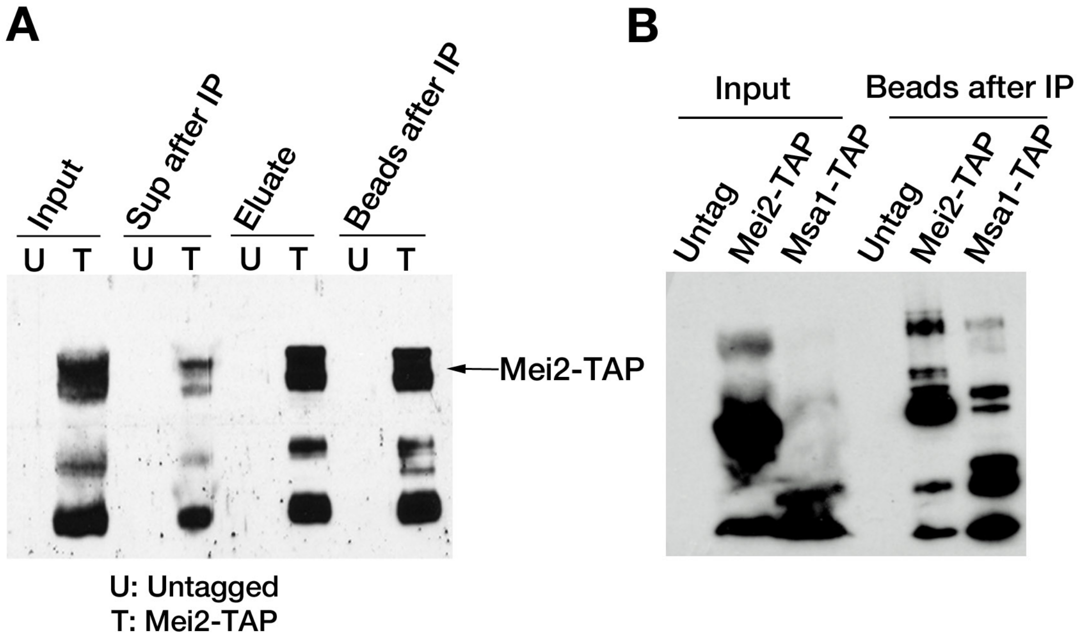

Supplementary Figure S3

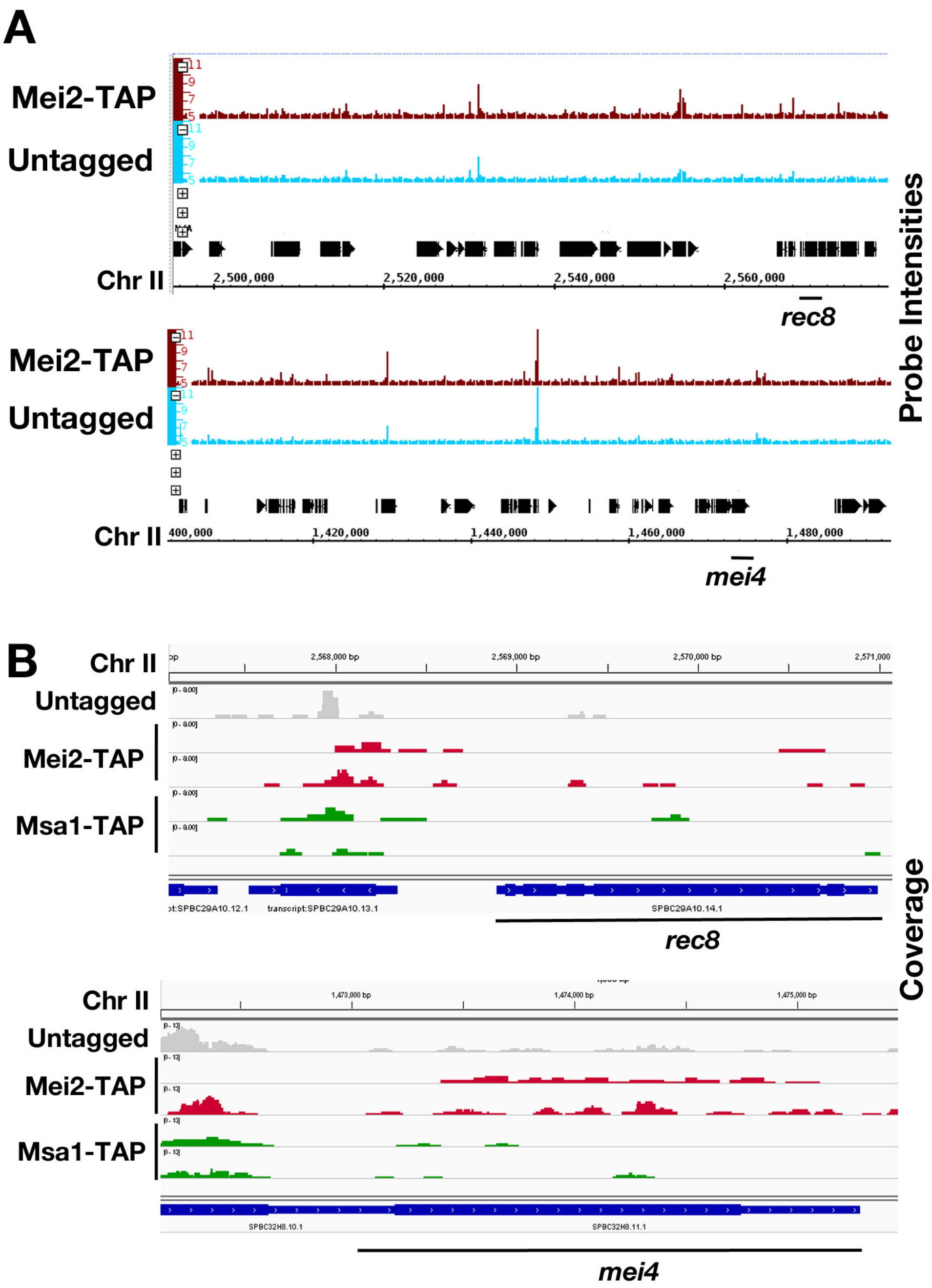

Supplementary Figure S4

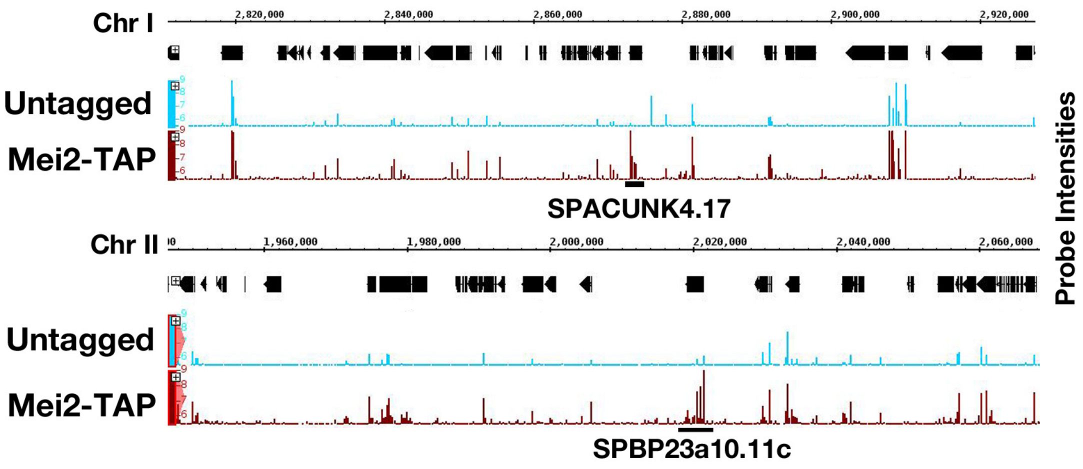

Supplementary Figure S5

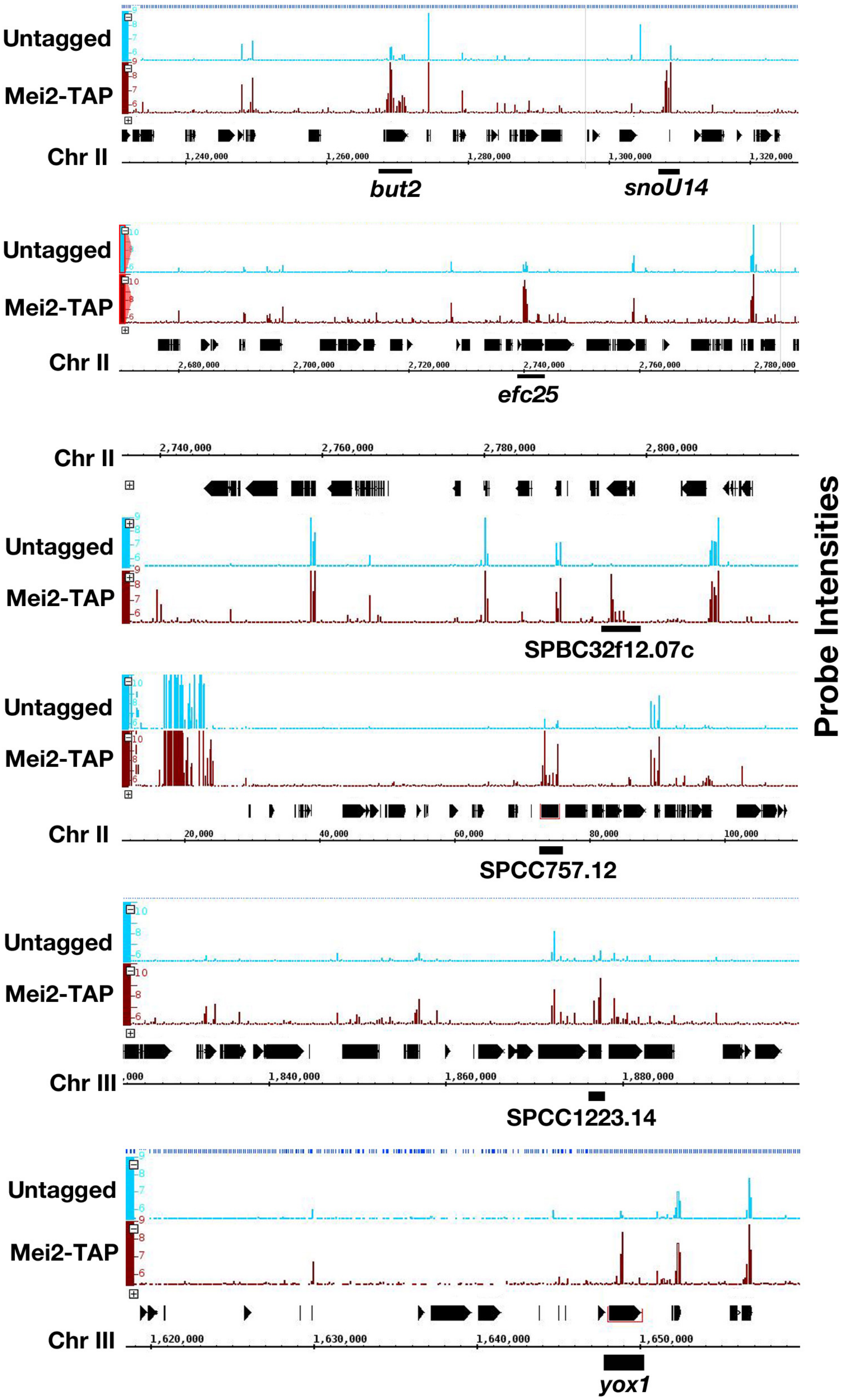

Supplementary Figure S6

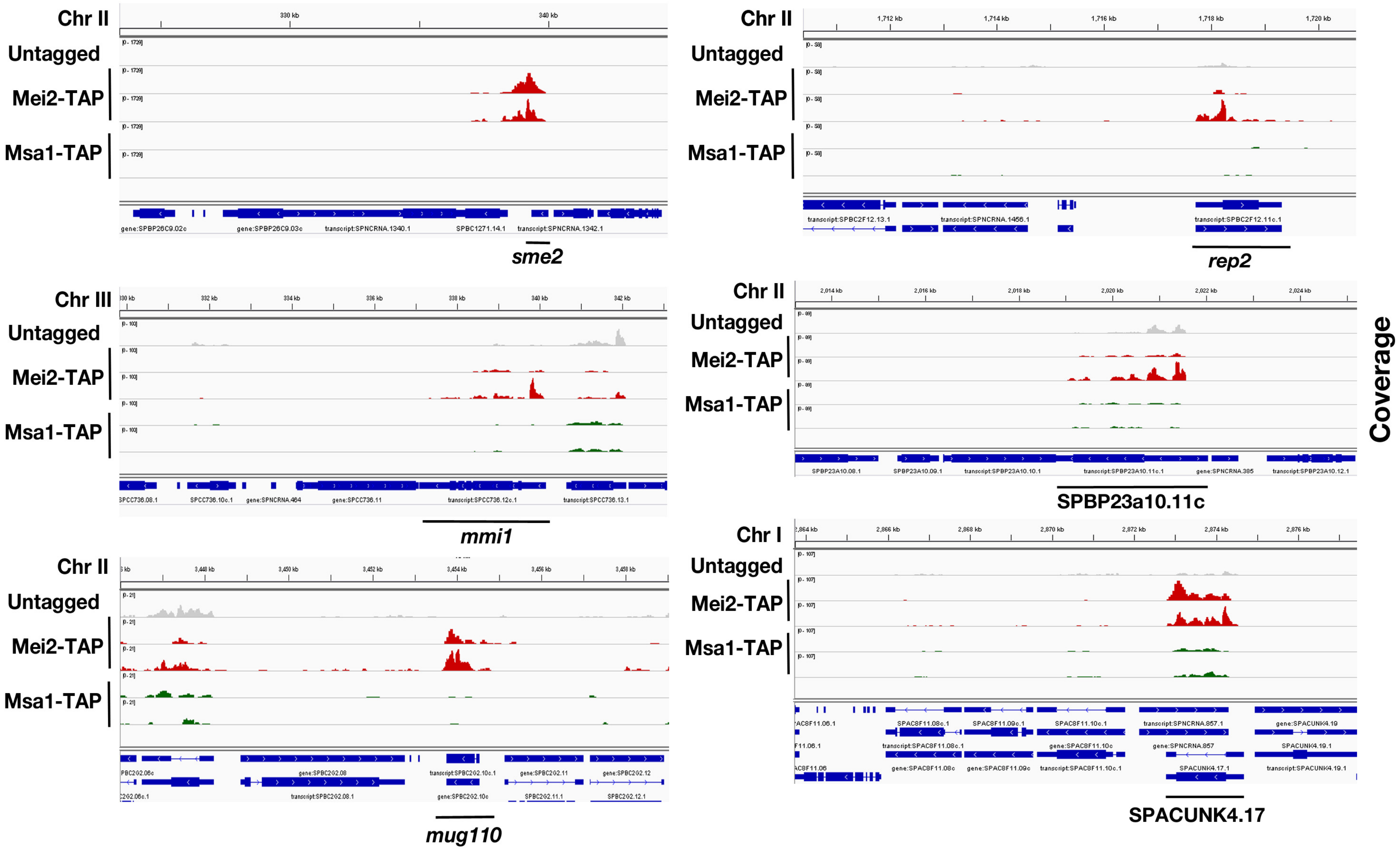

Supplementary Figure S7

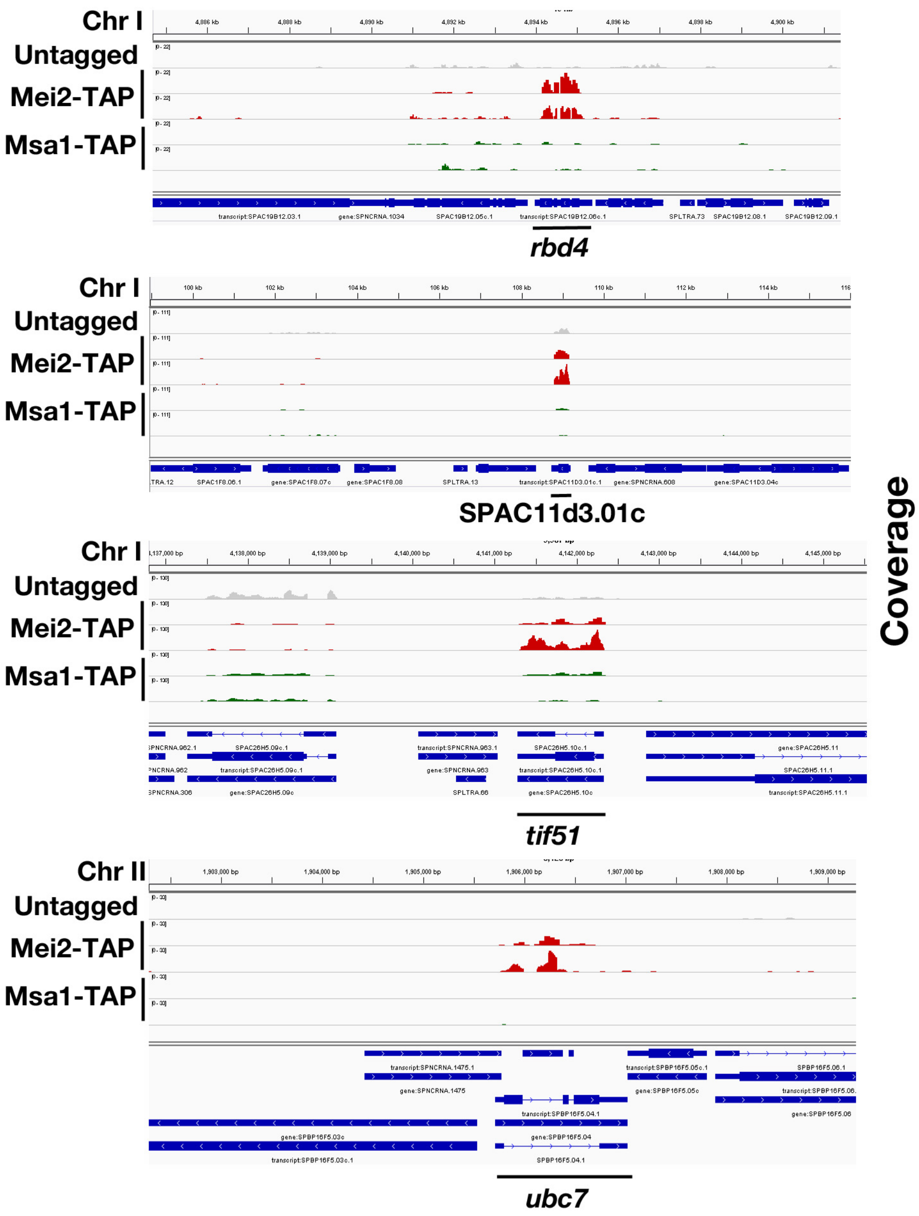

Supplement: Supplementary Figures [file rsob180110supp1.pdf]
