# Supplementary material for: mmi1 and rep2 mRNAs are novel RNA targets of the Mei2 RNA-binding protein during early meiosis in Schizosaccharomyces pombe
Source: Open Biol. 2018 Sep 26;8(9):180110. doi: 10.1098/rsob.180110 (PMC6170507; doi:10.1098/rsob.180110)
Supplement: Supplementary Legends [file rsob180110supp2.pdf]

Supplementary Figure Legends

Supplementary Figure S1. Mei2-TAP and Msa1-TAP expression during meiosis. (A) Western blot showing the expression of Mei2-TAP in an h^{90} strain after nitrogen starvation in ME media. Full-length Mei2-TAP expression is seen between 3-6 hours in this experiment. (B) Western blot showing Msa1-TAP expression in an h^{90} strain after nitrogen starvation in ME. Full-length Msa1-TAP is seen throughout meiosis. (C) Western blot showing Mei2-TAP and Msa1-TAP protein expression after adding increased amounts of protease inhibitor cocktail to the lysis buffer.

Supplementary Figure S2. Immunoprecipitation of Mei2-TAP and Msa1-TAP. (A) Western blot showing enrichment of Mei2-TAP after immunoprecipitation with IgG-agarose beads, and elution. (B) Western blot showing Mei2-TAP and Msa1-TAP enrichment after UV crosslinking and immunoprecipitation using IgG-agarose beads. 1:5000 PAP antibody was used to detect TAP-tagged proteins.

Supplementary Figure S3. Non-targets of Mei2, *rec8* and *mei4*. (A) Probe intensities from Mei2-TAP RIP-CHIP and (B) CLIP-Seq coverage from Mei2-TAP and Msa1-TAP CLIP-Seq of *rec8* and *mei4* loci showing that these RNAs are not enriched. Y-axes are group-autoscaled.

Supplementary Figure S4. High-confidence RNA targets of Mei2 determined by RIP-CHIP with uncertain meiotic roles. (A) IGB view from RIP-CHIP showing the enrichment at the SPACUNK4.17 and SPBP23a10.11c loci. These genes have uncertain roles in meiosis. Y-axes are group-autoscaled.

Supplementary Figure S5. RIP-CHIP only targets of Mei2. IGB view of RNAs enriched only in RIP-CHIP, but not in CLIP-Seq. Y-axes are group-autoscaled.

Supplementary Figure S6. CLIP-Seq enrichments of high-confidence RNA targets of Mei2. IGV snapshots of enrichments at loci determined as high-confidence Mei2 targets. For Mei2-TAP and Msa1-TAP, CLIP-Seq enrichments from biological replicates are shown. Y-axes are group-autoscaled.

Supplementary Figure S7. CLIP-Seq only targets of Mei2. IGV snapshots showing enrichments of Mei2 targets determined by CLIP-Seq but not RIP-CHIP. Y-axes are group-autoscaled.
